# Complete Mitogenomes of Three *Hyphessobrycon* (Characiformes, Characidae) Species and Phylogenetic Analysis of Characidae

**DOI:** 10.3390/ani16152415

**Published:** 2026-08-05

**Authors:** Yu-Zhe He, Zhi-Peng Guo, Zhi-Wei Huang, Wei-Jie Li, Huai-Yang Fang, Zong-Yao Zhang, Xian-Ru Li, Cheng-He Sun, Yang-Liang Gu

**Affiliations:** 1 National Key Laboratory of Water Environmental Simulation and Pollution Control, South China Institute of Environmental Sciences, Ministry of Ecology and Environment, Guangzhou 510530, China; 2 Co-Innovation Center for Sustainable Forestry in Southern China, College of Life Sciences, Nanjing Forestry University, Nanjing 210037, Chinasunchenghe@njfu.edu.cn (C.-H.S.)

**Keywords:** neotropics, genetic feature, taxonomic, phylogenetic relationship, freshwater fish

## Abstract

Advances in biotechnological tools have accelerated the genomic characterization of non-model organisms, providing insights for broader bioprospecting efforts. In this context, *Hyphessobrycon*, one of the most species-rich and widely distributed genera in the family Characidae, remains largely unexplored at the mitogenomic level. We sequenced the complete mitogenomes of three *Hyphessobrycon* species, compared them with those of other *Hyphessobrycon* species, and conducted a phylogenetic analysis. We found that *Hyphessobrycon* mitogenomes are similar in terms of structure and size, codons with relative synonymous codon usage >1 usually end with A or T, and the genus *Hyphessobrycon* is polyphyletic. Our study provides additional evidence for the phylogenetic relationships in Characidae; however, mitogenomes of more *Hyphessobrycon* species need to be characterized in future studies.

## Simple Summary

This study characterized the complete mitochondrial genomes of three *Hyphessobrycon* species (*H. myrmex*, *H. psittacus*, *H. procyon*), compared them with those of other *Hyphessobrycon* species, and conducted a phylogenetic analysis. In the phylogenetic analysis, not all *Hyphessobrycon* species were classified together. Hence, the genus *Hyphessobrycon* is polyphyletic, as supported by the findings of previous studies. This polyphyly can be attributed to biogeographical factors, such as vicariance events or a history of physical alterations and changes in connectivity between the basins that make up the Amazon basin. We also found that species belonging to different genera, such as *H. herbertaxelrodi* and *H. armstrongi*, are more closely related than among *Hyphessobrycon* species themselves. More mitogenome sequences of *Hyphessobrycon* species are expected to be characterized in the future and will aid in further studies.

## 1. Introduction

The neotropics are home to the world’s most diverse assemblage of freshwater fishes; however, the phylogenetic relationships and species delineations among neotropical freshwater fishes, particularly at recent time scales, remain uncertain [1,2,3]. Characidae is one of the most diverse neotropical fish families, with approximately 163 genera and over 1150 identified species [4]. *Hyphessobrycon* is one of the most species-rich and widely distributed genera in the family Characidae, comprising over 160 species distributed from Veracruz, Mexico, to Mar Chiquita Lagoon in Buenos Aires, Argentina [5,6,7,8,9,10,11,12,13]. Recent studies on *Hyphessobrycon* species have contributed to ecotoxicology [14] and provided new insights into copper toxicology [15].

Morphology-based classification of Characidae is difficult because of the morphological similarities among numerous species [3,16,17,18]. Therefore, mitochondrial genes are being increasingly used to investigate phylogenetic relationships within Characidae [19,20,21], necessitating comprehensive comparisons of the morphological and genetic features of various Characidae species [21].

Recent studies have described novel and rediscovered *Hyphessobrycon* species [22,23]; however, only eight complete mitogenomes of *Hyphessobrycon* species have been reported to date [24,25,26]. To address this knowledge gap, we sequenced the mitogenomes of three *Hyphessobrycon* species, *H. myrmex*, *H. psittacus*, and *H. procyon*, and compared them with those of 41 other Characiformes species. In addition, we conducted a phylogenetic analysis of Characidae species. Our aim was to expand the mitogenome database of *Hyphessobrycon* species and provide additional evidence for the phylogenetic relationships within Characidae.

## 2. Materials and Methods

### 2.1. Experimental Materials and DNA Extraction

Specimens of the three *Hyphessobrycon* species (*H. myrmex*, *H. psittacus*, and *H. procyon*) used in this study were sourced from a local bird, fish, and insect market. DNA was extracted from tail fin tissues and assessed for quality using 1% agarose gel electrophoresis and a NanoDrop 2000 instrument (Thermo Fisher Scientific, Waltham, MA, USA).

### 2.2. Genome Sequencing, Assembly, and Annotation

Complete mitogenomes were sequenced using the Illumina NovaSeq 6000 platform in 150-bp paired-end read mode at Personalbio (Shanghai, China). Raw reads were screened and assembled into complete mitogenomes using Lasergene 7.1 (DNAStar, Madison, WI, USA). Geneious v2024.0.5 [27] was used to identify the start and stop codons of protein-coding genes (PCGs) and MEGA7 [28] was used to compare the assembled mitogenomes with those of closely related species.

### 2.3. Sequence Analysis

Mitogenome sequences of eight *Hyphessobrycon* species were downloaded from the National Center for Biotechnology Information (NCBI) website (Table 1). PhyloSuite v1.2.2 [29] was used to extract PCGs, transfer (t)RNA genes, ribosomal (r)RNA genes, and non-coding control regions (CRs) from the mitogenomes and to investigate the nucleotide composition, conventional start and stop codons, relative synonymous codon usage (RSCU), and amino acid usage. The ratio of the non-synonymous substitution rate (Ka) to the synonymous substitution rate (Ks) for all PCGs of seven *Hyphessobrycon* mitogenomes and the nucleotide diversity of all PCGs of 11 *Hyphessobrycon* mitogenomes were determined using MEGA7 [28] and DnaSP v6 [30], respectively, and plotted using https://www.bioinformatics.com.cn (accessed on 10 December 2024). A PCG cluster plot was created using Chiplot (https://www.chiplot.online/; accessed on 7 August 2022).

### 2.4. Phylogenetic Analysis

Mitogenome sequences of 41 species from 19 genera of the order Characiformes were downloaded from the NCBI website (Table 1) and used, along with the three mitogenomes sequenced in this study, to construct phylogenetic trees. *Lebiasina astrigata* was used as an outgroup. PhyloSuite v1.2.2 [29] was used to extract the PCGs, tRNAs, rRNAs, and non-coding CRs.

Multiple sequence alignments were generated using MAFFT v7.313 [31] and MACSE v1.03 [32], and unreliable or poorly aligned regions were removed using Gblocks 0.91b and trimAl v1.2. ModelFinder [33] was used to concatenate the PCG and tRNA sequences and select the optimal partitioning scheme and evolutionary model. Maximum likelihood (ML) phylogenies were inferred using IQ-TREE v1.6.8 [34] with the edge-linked partition model for 5000 ultrafast bootstraps [35] and the Shimodaira–Hasegawa-like approximate likelihood ratio test [36]. Bayesian inference (BI) phylogenies were inferred using MrBayes 3.2.6 [37] with the partition model (two parallel runs, 10,000 generations), and the first 25% of the sampled data was discarded as burn-in. ML and BI trees were visualized and beautified using Interactive Tree Of Life (http://itol.embl.de).

## 3. Results

### 3.1. Mitogenome Organization and Composition in Three Hyphessobrycon Species

**Figure 1:**
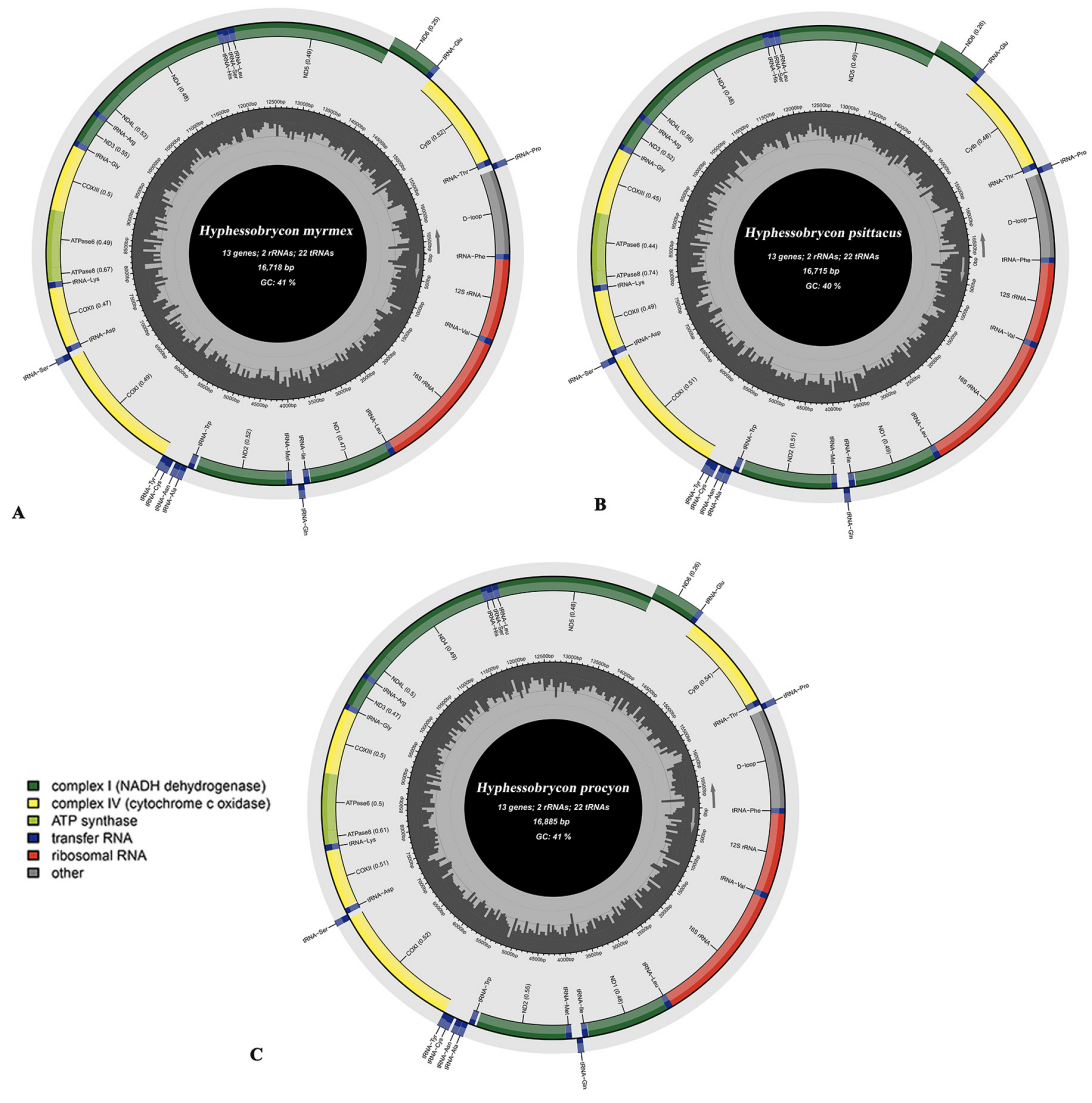
Mitogenome organization in *H. myrmex* (**A**), *H. psittacus* (**B**), and *H. procyon* (**C**).

The complete circular mitogenomes of *H. myrmex*, *H. psittacus*, and *H. procyon* are double-stranded molecules of length 16,718, 16,715, and 16,885 bp, respectively (Figure 1). The three mitogenomes comprise all 37 typical mitochondrial genes (13 PCGs, 22 tRNAs, and 2 rRNAs) and 1 non-coding CR. Their structures and sizes are similar to those of other *Hyphessobrycon* species, including *H. erythrozonus* (16,710 bp), *H. heterorhabdus* (17,021 bp), and *H. pulchripinnis* (17,020 bp) [24,25,26,38].

**Figure 2:**
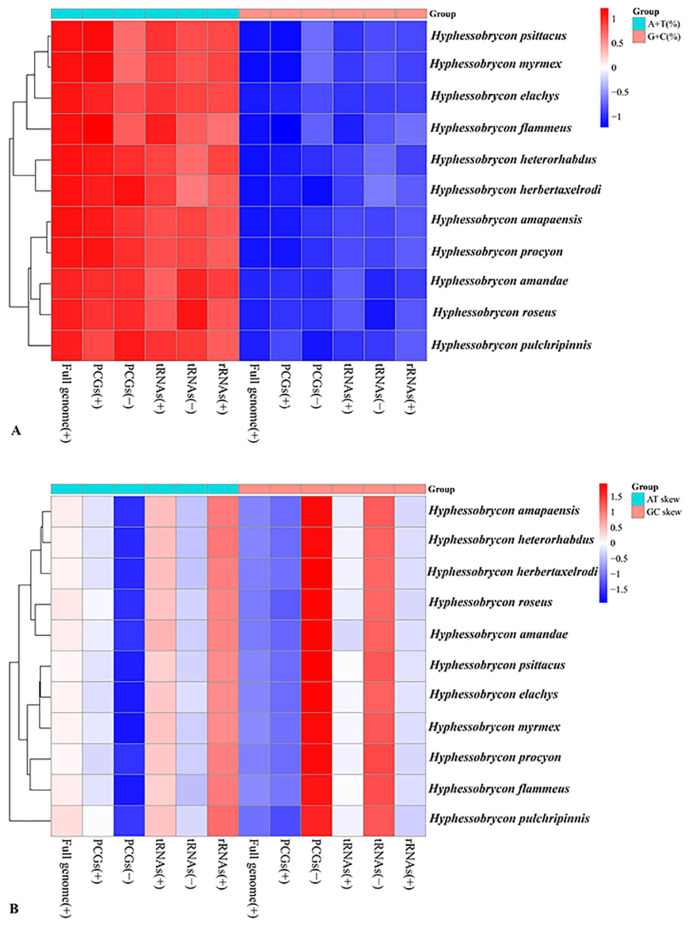
Nucleotide composition of 11 complete *Hyphessobrycon* mitogenomes based on A + T and C + G contents (**A**) and AT and GC skew (**B**). “+” and “−” indicate genes located on the heavy (H) and light (L) strands, respectively.

The nucleotide composition of 11 complete *Hyphessobrycon* mitogenomes was investigated by calculating the A + T and C + G contents (Figure 2A), as well as the AT skew and GC skew (Figure 2B). The A + T contents in the entire genomes (+), PCGs, tRNAs, and rRNAs (+) of the 11 mitogenomes were positive, whereas the C + G contents were negative. Similarly, the AT skew was largely positive in the entire genomes (+), tRNAs (+), and rRNAs (+) of the 11 mitogenomes, whereas the GC skew was negative. However, in PCGs (+) of the 11 complete mitogenomes, both the AT skew and the GC skew were negative, whereas in PCGs (−) of all 11 mitogenomes, the AT skew was negative and the GC skew was positive.

The 22 tRNAs are discontinuously distributed throughout the three mitogenomes. The total length of the tRNAs is 1552 bp in all three species, accounting for 9.3% (*H. myrmex*), 9.3% (*H. psittacus*), and 9.2% (*H. procyon*) of the entire mitogenome. The lengths of the 22 tRNAs range from 67 to 75 bp. The two rRNA genes are encoded on the H-strand in all three species, and the 16S rRNA gene is flanked by the tRNA-Val and tRNA-Leu genes, whereas the 12S rRNA gene is flanked by the tRNA-Phe and tRNA-Val genes. The 16S rRNA gene length ranges from 1668 to 1672 bp, and the 12S rRNA gene length ranges from 943 to 949 bp in the three mitogenomes. The D-loop region flanked by tRNA-Phe and tRNA-Pro is 988 bp in *H. myrmex*, 983 bp in *H. psittacus*, and 1007 bp in *H. procyon*.

We identified multiple overlaps between the contiguous genes. The *H. myrmex* and *H. psittacus* mitogenomes have 14 overlapping regions, ranging from 1 to 67 bp, whereas those of *H. procyon* have 13 overlapping regions, ranging from 1 to 50 bp. The longest overlap in the three mitogenomes was found between tRNA-Gln and tRNA-Met, ranging from 54 to 67 bp. In addition, seven spacer regions were detected in all three mitogenomes, with a total length of 38 bp. The spacer positions and sizes are relatively consistent among the three species.

### 3.2. PCGs and Codon Usage

The total length of PCGs in the 11 *Hyphessobrycon* species ranges from 11,412 bp (*H. amandae*) to 11,457 bp (*H. herbertaxelrodi* and *H. amapaensis*). Most PCGs have the conventional start codon ATG, but some start with GTG, ATA, ATT, TTG, CTG, or AGT. In the three newly sequenced mitogenomes, only *COXI* starts with GTG in all three species, and *ATPase6* starts with TTG in *H. psittacus* and *H. procyon* and CTG in *H. myrmex*. Among the 11 *Hyphessobrycon* species, PCGs have the termination codons TAA, TAG, and AGG and the incomplete codons TA and T. In all mitogenomes, the termination codon TAA occurs at a higher frequency than the other four termination codons, whereas the stop codon TA is the least frequent codon. In the three newly sequenced mitogenomes, the stop codons of the corresponding PCGs are largely the same, except for *Cytb*, which terminates with T in the *H. procyon* mitogenome.

**Figure 3:**
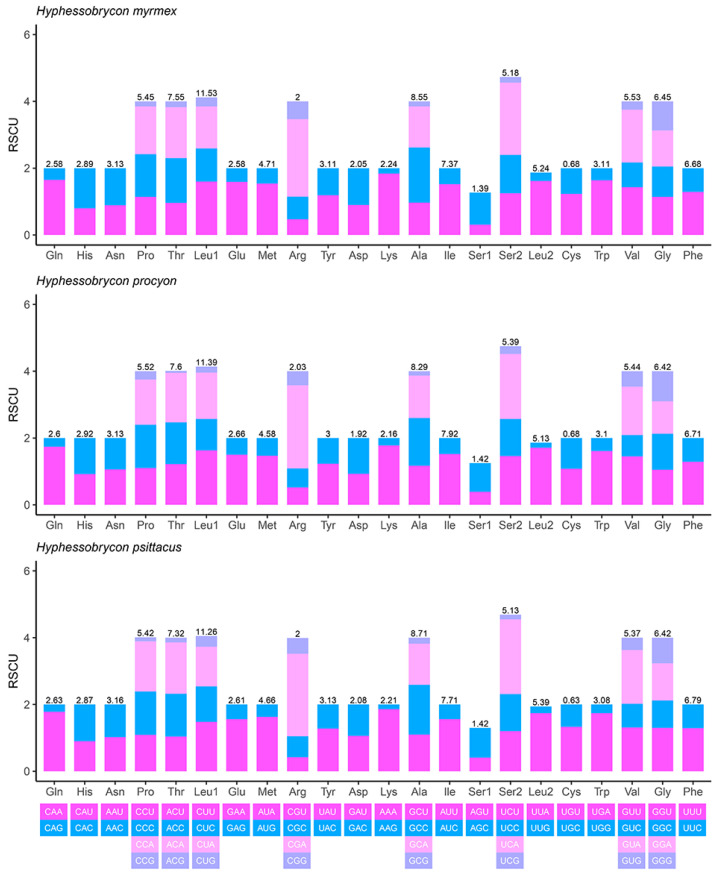
Relative synonymous codon usage (RSCU) for the 13 protein-coding genes (PCGs) in the three sequenced *Hyphessobrycon* mitogenomes. The frequency of use of each amino acid is shown above the stacked columns.

**Figure 4:**
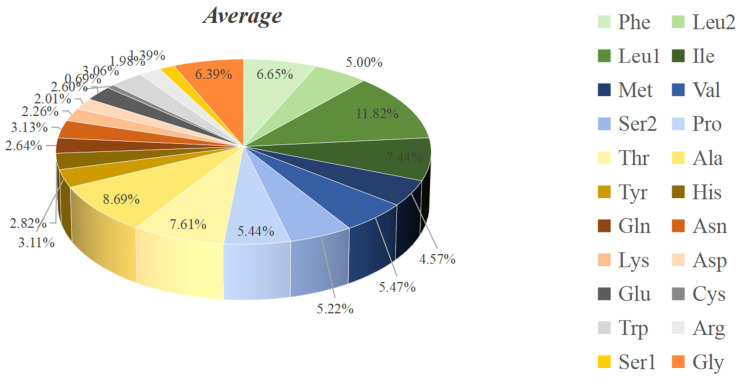
Average amino acid frequencies in the mitogenomes of 11 *Hyphessobrycon* species.

The RSCU and amino acid usage in the 11 complete *Hyphessobrycon* mitogenomes are illustrated in Figure 3 and Figure 4. The two most-used amino acids are Leu (12.91%) and Ala (9.04%), whereas Cys is the least used (0.68%). Codons with RSCU > 1 usually end with A or T, as previously reported by Xu et al. [38].

**Figure 5:**
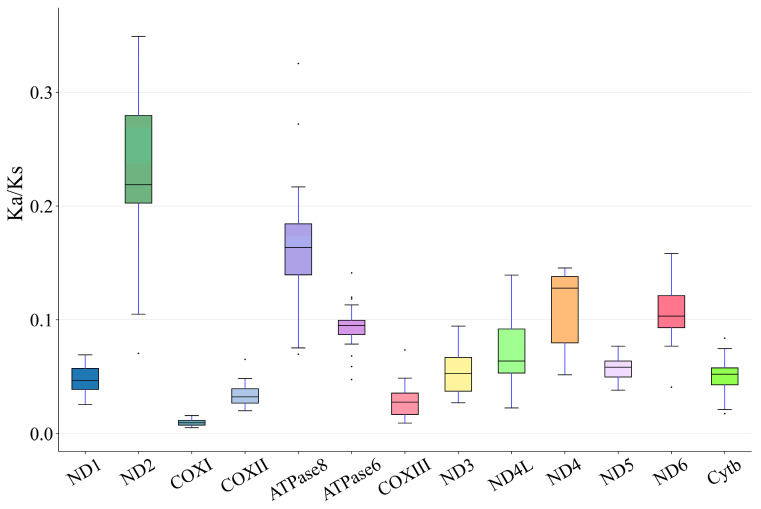
Ratio of the non-synonymous substitution rate to the synonymous substitution rate (Ka/Ks) for the 13 PCGs in seven *Hyphessobrycon* mitogenomes.

**Figure 6:**
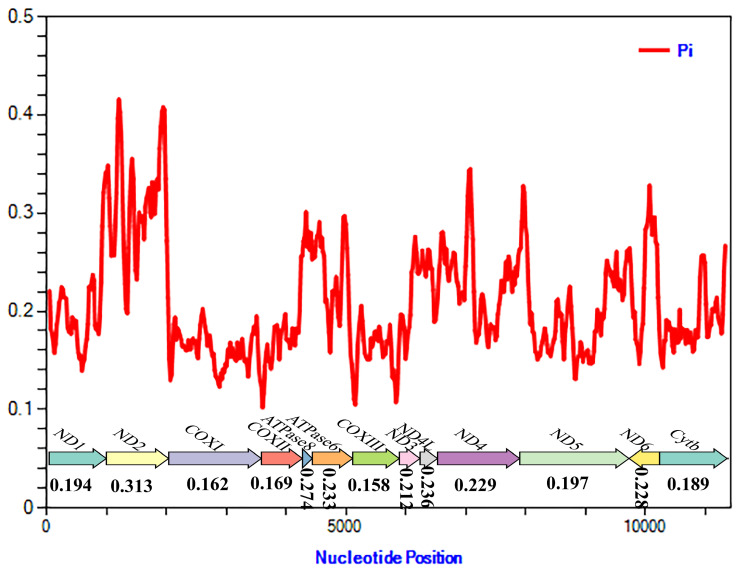
Nucleotide diversity of the 13 PCGs of 11 *Hyphessobrycon* mitogenomes. The red curve represents nucleotide diversity (Pi); the average value for each gene is displayed below the gene name.

The Ka/Ks ratios for all PCGs of the seven *Hyphessobrycon* mitogenomes are shown in Figure 5. *ND2* had the highest average Ka/Ks value (0.237), which was <1, as were those of the other PCGs. *ND2* also showed the largest difference in Ka/Ks values among the seven *Hyphessobrycon* mitogenomes. The nucleotide diversity of all PCGs in the 11 *Hyphessobrycon* mitogenomes is shown in Figure 6. The highest nucleotide diversity was observed in *ND2* (0.313), whereas *COXIII* (0.158) and *COXI* (0.162) had the lowest nucleotide diversity.

### 3.3. Phylogenetic Analysis

**Figure 7:**
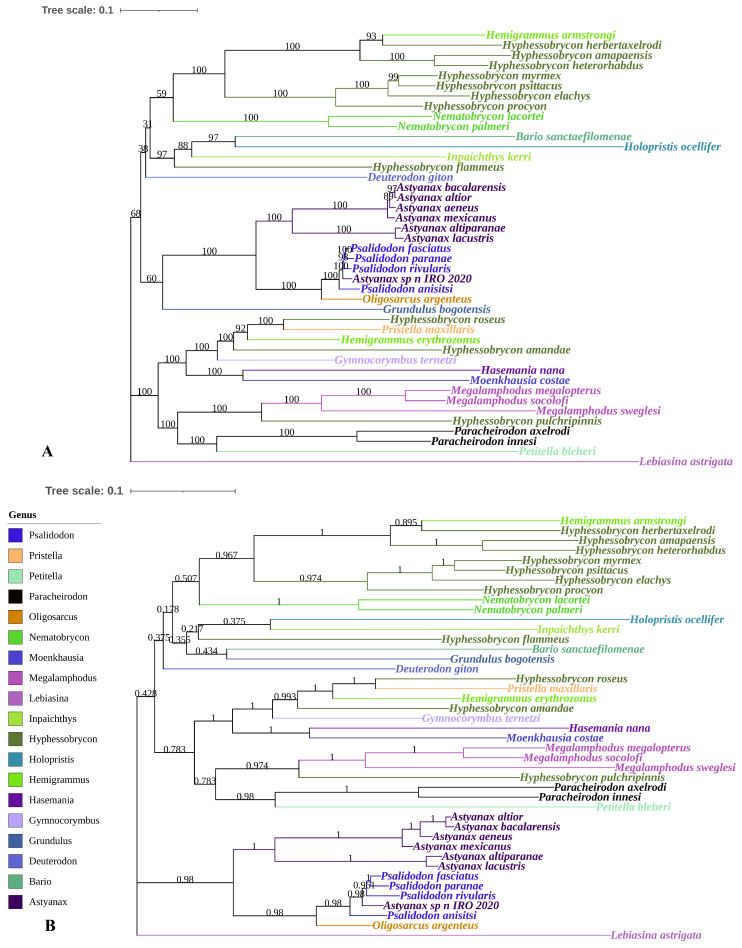
Maximum likelihood (ML) (**A**) and Bayesian inference (BI) (**B**) phylogenetic trees based on PCG, transfer (t)RNA, and ribosomal (r)RNA sequences of 44 species.

We generated two phylogenetic trees using the ML and BI methods, including sequences from 43 Acestrorhamphidae species and one Lebiasinidae species, *L. astrigata*, used as an outgroup (Figure 7). Nearly all *Astyanax* species clustered together; however, *Astyanax* sp. *IRO-2020* was designated as a sister group to *Psalidodon* species. Seven *Hyphessobrycon* species were classified with *Hemigrammus armstrongi*, whereas four other *Hyphessobrycon* species were not classified in this group (e.g., *H. pulchripinnis* was classified with *Megalamphodus* species), indicating that the genus *Hyphessobrycon* is polyphyletic. *Hyphessobrycon heterorhabdus* was found to be in close phylogenetic proximity to *H. amapaensis*, *H. psittacus* to *H. myrmex*, and *H. herbertaxelrodi* to *H. armstrongi*.

## 4. Discussion

The mitogenomes of *Hyphessobrycon* species, including the three species sequenced in this study, are similar in structure and size [24,25,26,38]. The three current mitogenomes have relatively high A + T content and low C + G content, consistent with those in previously reported *Hyphessobrycon* mitogenomes [24,25,38]. The GC skew values were low in the three complete mitogenomes, indicating a GC bias, as observed in other *Hyphessobrycon* fish mitogenomes [25,26,38].

Among the 11 *Hyphessobrycon* species, most PCGs have the conventional start and stop codons, ATG and TAA. Among teleosts, including the three species sequenced here, the incomplete codons TA and T occur but may be completed to TAA via poly-A tail addition during RNA processing [25,39,40]. *ND2* has the highest average Ka/Ks and nucleotide diversity, whereas *COXI* has the lowest average Ka/Ks and nucleotide diversity. Hence, these genes may serve as molecular markers, as previously reported by Sun et al. [25].

In the phylogenetic analysis, not all *Hyphessobrycon* species were classified together. Hence, the genus *Hyphessobrycon* is polyphyletic, as supported by the findings of previous studies [6,21,25,38,41]. This polyphyly can be attributed to biogeographical factors, such as vicariance events or a history of physical alterations and changes in connectivity between the basins that make up the Amazon basin [24,40,42].

We also found that species belonging to different genera, such as *H. herbertaxelrodi* and *H. armstrongi*, are more closely related than *Hyphessobrycon* species among themselves [38]. Xu et al. [26] reported large variability in morphological characteristics among species of the same genus, whereas species from different Characiformes genera had similar morphological characteristics. More mitogenome sequences of *Hyphessobrycon* species are expected to be characterized in the future and will aid in further studies.

## 5. Conclusions

This study characterized the complete mitochondrial genomes of three *Hyphessobrycon* species, compared them with those of other *Hyphessobrycon* species, and conducted a phylogenetic analysis. We found that *Hyphessobrycon* mitogenomes are similar in terms of structure and size, codons with relative synonymous codon usage >1 usually end with A or T, and the genus *Hyphessobrycon* is polyphyletic. Our study provides additional evidence for the phylogenetic relationships in Characidae; however, mitogenomes of more *Hyphessobrycon* species need to be characterized in future studies.

## Figures and Tables

**Table 1:** Mitogenomes of the Acestrorhamphidae and Lebiasinidae species (outgroup) used in the phylogenetic analysis.

Order	Family	Genus	Species	GenBank No.	Full Length (bp)
Characiformes	Acestrorhamphidae	*Psalidodon*	*Psalidodon rivularis*	MT428070.1	16,812
			*Psalidodon paranae*	NC_031380.1	16,707
			*Psalidodon fasciatus*	OP218849.1	16,811
			*Psalidodon anisitsi*	NC_066994.1	16,920
		*Pristella*	*Pristella maxillaris*	NC_066992.1	16,896
		*Petitella*	*Petitella bleheri*	NC_027946.1	17,021
		*Paracheirodon*	*Paracheirodon innesi*	KT783482.1	16,962
			*Paracheirodon innesi*	NC_028279.1	16,759
			*Paracheirodon axelrodi*	AB898197.1	17,100
		*Oligosarcus*	*Oligosarcus argenteus*	NC_044969.1	16,711
		*Nematobrycon*	*Nematobrycon palmeri*	NC_051983.1	17,340
			*Nematobrycon lacortei*	PP760379.2	17,585
		*Moenkhausia*	*Moenkhausia costae*	MZ364159.1	16,560
		*Megalamphodus*	*Megalamphodus sweglesi*	MW315751.1	16,080
			*Megalamphodus socolofi*	NC_066990.1	17,132
			*Megalamphodus megalopterus*	NC_053878.1	16,773
		*Inpaichthys*	*Inpaichthys kerri*	NC_057167.1	17,032
		*Hyphessobrycon*	*Hyphessobrycon roseus*	MW315749.1	17,046
			*Hyphessobrycon pulchripinnis*	MW331227.1	17,618
			*Hyphessobrycon psittacus*	PV998959	16,715
			*Hyphessobrycon procyon*	PV564007	16,885
			*Hyphessobrycon myrmex*	PV992874	16,718
			*Hyphessobrycon heterorhabdus*	NC_080887.1	17,021
			*Hyphessobrycon herbertaxelrodi*	NC_050876.1	17,417
			*Hyphessobrycon flammeus*	MW315748.1	16,008
			*Hyphessobrycon elachys*	MW315747.1	17,224
			*Hyphessobrycon amapaensis*	NC_066989.1	17,824
			*Hyphessobrycon amandae*	MT484069.1	16,701
		*Holopristis*	*Holopristis ocellifer*	NC_066993.1	18,141
		*Hemigrammus*	*Hemigrammus erythrozonus*	MT484070.1	16,710
			*Hemigrammus armstrongi*	NC_066991.1	16,789
		*Hasemania*	*Hasemania nana*	NC_022724.1	16,581
		*Gymnocorymbus*	*Gymnocorymbus ternetzi*	MZ363625.1	17,999
		*Grundulus*	*Grundulus bogotensis*	NC_026195.1	17,123
		*Deuterodon*	*Deuterodon giton*	NC_044970.1	16,643
		*Bario*	*Bario sanctaefilomenae*	NC_056781.1	18,437
		*Astyanax*	*Astyanax* sp. IRO-2020	MT428068.1	16,714
			*Astyanax mexicanus*	BK013062.1	16,768
			*Astyanax lacustris*	NC_053756.1	16,763
			*Astyanax bacalarensis*	PV765695.1	16,769
			*Astyanax altiparanae*	MN583176.1	16,730
			*Astyanax altior*	PV765696.1	16,766
			*Astyanax aeneus*	BK013055.1	16,769
	Lebiasinidae	*Lebiasina*	*Lebiasina astrigata*	MH921292.1	16,899

## Data Availability

The data presented in this study are openly available in the NCBI repository, accession numbers PV998959, PV564007, and PV992874.

## Abbreviations

The following abbreviations are used in this manuscript:

BI	Bayesian inference
CR	Control region
ML	Maximum likelihood
NCBI	National Center for Biotechnology Information
Ka	Non-synonymous substitution rate
PCG	Protein-coding gene
RSCU	Relative synonymous codon usage
rRNA	Ribosomal RNA
Ks	Synonymous substitution rate
tRNA	Transfer RNA
